# Perceptions of control over different causes of death and the accuracy of risk estimations

**DOI:** 10.1007/s10389-023-01910-8

**Published:** 2023-04-14

**Authors:** Richard Brown, Elizabeth Sillence, Gillian Pepper

**Affiliations:** Psychology Department, Northumbria University, Northumberland Building, Newcastle, NE1 8SG UK

**Keywords:** Risk perceptions, Health perceptions, Health behaviours, Avoidable death, Public health, Health psychology

## Abstract

**Background:**

A large number of deaths could be avoided by improving health behaviours. The degree to which people invest in their long-term health is influenced by how much they believe they can control their risk of death. Identifying causes of death believed to be uncontrollable, but likely to occur, may provide actionable targets for health interventions to increase control beliefs and encourage healthier behaviours.

**Method:**

We recruited a nationally representative online sample of 1500 participants in the UK. We assessed perceived control, perceived personal likelihood of death, certainty of risk estimation, and perceived knowledge for 20 causes of death. We also measured overall perceived uncontrollable mortality risk (PUMR) and perceived prevalence for each of the Office for National Statistics’ categories of avoidable death.

**Findings:**

Risk of death due to cancer was considered highly likely to occur but largely beyond individual control. Cardiovascular disease was considered moderately controllable and a likely cause of death. Drugs and alcohol were perceived as risks both high in control and low in likelihood of death. However, perceptions of control over specific causes of death were found not to predict overall PUMR, with the exception of cardiovascular disease. Finally, our sample substantially overestimated the prevalence of drug and alcohol-related deaths in the UK.

**Conclusions:**

We suggest that more can be done by public health communicators to emphasise the lifestyle and behavioural changes that individuals can make to reduce their general cancer risk. More work is needed to understand the barriers to engaging with preventative behaviours and maintaining a healthy heart. Finally, we call for greater journalistic responsibility when reporting health risks to the public.

**Supplementary Information:**

The online version contains supplementary material available at 10.1007/s10389-023-01910-8.

## Introduction

Approximately 23% of deaths in the UK in 2020 were considered avoidable, many of them being due to diseases often associated with unhealthy behaviours (e.g., cardiovascular disease, respiratory disease, and illnesses related to drug and alcohol use (Office for National Statistics 2022). The amount of effort that people invest in their long-term health and safety varies considerably between individuals. Therefore, understanding the factors that drive this variation in health effort may help to inform interventions aimed at reducing avoidable deaths.

Previous research suggests that the degree to which we invest in our long-term health is influenced by how much we believe we can control our risk of death. Perceived uncontrollable mortality risk (PUMR) is that portion of our risk of death which we believe cannot be mitigated by health effort (Nettle 2010; Pepper and Nettle 2014b; Pepper and Nettle 2017a, b; Brown et al. 2021a). The Uncontrollable Mortality Risk Hypothesis (UMRH) states that those who believe they are more likely to die as a result of factors that are beyond their control should be less motivated to engage in positive health behaviours (Pepper and Nettle 2014a; Brown and Pepper 2023). The UMRH is supported by a growing body of theoretical, correlational and experimental evidence (Nettle 2010; Pepper and Nettle 2014b, a; Brown et al. 2021a, b; Brown et al. 2022). For example, Pepper and Nettle (2014b) found that the effect of lower socioeconomic status on reported health effort was mediated by PUMR. More recent research during the COVID-19 pandemic found that PUMR was associated with reduced adherence to guidelines on preventative health behaviours relating to diet, exercise and smoking (Brown et al. 2021a). Further study of PUMR and its driving factors may therefore be fundamental to understanding differences in health behaviours which may, in turn, help to reduce rates of avoidable death (Brown and Pepper 2022).

Given the relationship between perceived control and health behaviour, understanding the specific perceptions that drive overall levels of PUMR may help to create interventions aimed at improving modifiable health behaviours. For example, do beliefs about the controllability of death due to cancer, cardiovascular disease or substance abuse have greater influence over PUMR compared to perceived control over environmental risks such as traffic accidents, exposure to violence or pollution? By identifying those risks widely believed to be uncontrollable, interventions that increase perceived control over these risks may help to improve associated health behaviours. Recent qualitative findings from a study exploring perceptions of control over different causes of death suggest differences between health conditions in the extent to which they are generally believed to be uncontrollable (Brown et al. 2022). For example, though dying from heart disease was broadly described as a controllable risk, the risk of dying from cancer was mostly considered to be uncontrollable. Furthermore, the qualitative findings showed that participants generally believed that traffic accidents and the effects of air pollution are potential causes of death that are personally uncontrollable due to uncertainty over the specific actions that could be taken to reduce risk. Investigating perceptions of control over mortality risk may help to inform targeted health interventions aimed at improving health behaviours associated with personally avoidable deaths. For example, if certain forms of cancer are widely believed to be beyond individual control, people may be less motivated to engage in the positive health behaviours likely to reduce their risk. In such cases, interventions tailored to emphasise the extent to which specific cancer risks are modifiable through behaviour may prove effective.

It is also important to consider the extent to which people are accurate in their estimations of the risks they face. Overestimating the likelihood of dying from certain causes of death may increase overall PUMR, potentially disincentivising preventative health behaviours. This may suggest the need for informational interventions to align perceptions of risk with objective exposures to encourage the public to respond more appropriately to the risks they face. Alternatively, where levels of risk associated with certain causes of death are accurately perceived by the public, research may look to identify the sources of risk that inform these beliefs to apply similar informational strategies to other causes of death. When studying perceptions of mortality risk, a *primary bias* has consistently been reported in which people typically overestimate rare risks to their health and underestimate common risks (Lichtenstein et al. 1978; Hakes and Viscusi 2004). For example, Slovic (1978) found that people typically overestimate their risk of dying as a result of homicide or natural disaster, but typically underestimate the likelihood of dying due to diabetes, cancer or stroke. Studying the perceived prevalence of different mortality risks may help to understand general beliefs about health and risk in the UK. However, to our knowledge, no study has compared the perceived prevalence of different leading causes of avoidable death in the UK population with objective measures of risk. Investigating mortality risk perceptions is of particular interest in response to potential changes to perceptions resulting from the COVID-19 pandemic. The perceived prevalence of common diseases may have been affected by the widely discussed neglect of major causes of death by governments during the COVID-19 pandemic (Holakouie-Naieni and Nematollahi 2020; Poorolajal 2020; Clerk 2021). Throughout the pandemic, concerns were raised regarding policies that saw the abrupt cessation of essential services to non-COVID patients, impacting patients’ ability to receive treatment for many common diseases (Poorolajal 2020). Risk of dying due to infectious disease is also likely to be particularly salient in the wake of COVID-19. Additionally, measuring perceptions of control associated with catastrophic risks such as those resulting from nuclear attack or climate change may also capture growing public concern regarding the conflict in Ukraine and the climate crisis (United Nations 2022; Drews et al. 2022). With respect to biases in perceptions of different causes of avoidable death, it is likely that the least common causes of avoidable death (accidental injuries, violence and infectious disease etc.,) will be overestimated, whereas the perceived prevalence of the most common causes of avoidable death (cancers, heart disease and respiratory disease) will be underestimated (Hakes and Viscusi 2004).

The aim of this study is to investigate perceptions of control over different causes of death in a nationally representative sample of the UK. Identifying the causes of death generally believed to be beyond individual control and investigating the extent to which specific perceptions of risk influence overall levels of PUMR will increase our understanding of the relationship between perceptions of control and health behaviours. Exploring the perceptual differences between a range of causes of death will help to identify targets for future health interventions directed at improving health behaviours and reducing rates of personally avoidable death.

## Methods

This study was approved by the Northumbria University research ethics system (ethical approval number 41708). Our measures, predictions and analytical plan are registered with the Open Science Framework [https://osf.io/dgwna].

Cognitive interviews were conducted to assess participants’ understanding of the questionnaire. Six participants were interviewed following recommendations for conducting cognitive interviews for refining survey items (Peterson et al. 2017). Pilot testing of the questionnaire was subsequently conducted to further develop the survey items and to test functionality. We recruited 39 participants for our pilot based on sample size recommendations for developing survey studies (Hertzog 2008). The refined questionnaire was presented to participants via the recruitment platform Prolific between 4-6 May 2022. Participants were paid incentive fees through this platform which were in line with the UK living wage (£8.91 per hour), in accordance with Northumbria University’s ethical guidelines for conducting research.

Our target sample size was 1500 participants based on recent guidance and practices set by YouGov and the Office for National Statistics (ONS) for surveying the opinions and perceptions of the UK public (Office for National Statistics 2021; YouGov 2021). Prolific provides a UK representative sample by screening participants based on age, gender and ethnicity in proportion to data derived from the UK’s 2021 census. From this sample, four participants were excluded due to technical errors during data collection, 24 participants were excluded due to inconsistencies between their Prolific profile information and their survey responses, five participants were excluded for taking over 90 minutes to complete the survey questionnaire, and those who reported the number of years in post-16 education as above 25 (*n* = 4) were removed as extreme outliers as their responses suggested a possible misinterpretation of the question. Our final sample comprised 1463 participants.

### Personal information

Personal information was recorded for participants’ age, gender and education. Discretionary income was measured by asking participants how much their household has each month to spend on non-essentials. Participants also provided scores for the Subjective Discretionary Income (SDI) scale (O'Guinn and Wells 1989; Rader et al. 2011). This scale asked participants the extent to which they agreed with three statements about the perceived ability of their household finances to satisfy their wants and needs on a five-point Likert scale ranging from ‘*strongly disagree*’ to ‘*strongly agree*’.

### Living and working environment

Participants were asked whether they believed their current neighbourhood is a safe place to live on a seven-point scale ranging from ‘*strongly disagree*’ to ‘*strongly agree*’ (An et al. 2017; Prins et al. 2013). Occupational exposure to risk was measured by using the Physical Working Environment Subscale from the European Working Conditions Survey 2015 (Eurofound 2017). Participants reported their exposure to 13 physical conditions of employment on a seven-point Likert scale ranging from ‘*never*’ to ‘*all of the time*’. These survey items included risks due to occupational activities that include maintaining tiring or painful positions, carrying or moving heavy loads, and being exposed to hazardous materials, high temperatures or noisy environments.

### Perceptions of risk

Participants provided a measure of perceived uncontrollable mortality risk (PUMR; Pepper and Nettle 2014b). Participants were asked to provide a score for their believed likelihood of living to the current average UK life expectancy at birth for their gender, provided they made the maximum effort to look after their health. Participants responded on a sliding scale from 0 *‘no chance’* to 100 ‘*certain*’. Responses were subtracted from 100 to provide PUMR scores which represent that portion of mortality risk which the participant believes is beyond their control.

Participants answered questions about their perceived control, personal likelihood of death, certainty of risk estimation, and perceived knowledge for 20 causes of death (see Table 1). These causes of death were selected from the ONS’ most common categories of avoidable deaths in the UK, public risks identified as most serious and likely by the UK National Risk Register 2020, as well as causes of death consistently highlighted by recent qualitative findings on perceptions of control over risk (Cabinet Office 2020; Office for National Statistics 2022; Brown et al. 2022). Participants were asked how much they thought they could control their own risk of dying from each cause of death on a sliding scale from 0 ‘*no control*’ to 100 ‘*complete control*’. Participants were also asked how likely they thought they were to die from each cause of death before the age of 81. Scores were provided on a sliding scale from 0 ‘*very unlikely*’ to 100 ‘*very likely*’. Participants’ certainty over their personal estimations of risk were then assessed on a sliding scale from 0 ‘*not at all sure*’ to 100 ‘*completely sure*’. Finally, participants were asked how knowledgeable they thought they were about each cause of death on a sliding scale from 0 ‘*not at all knowledgeable*’ to 100 ‘*extremely knowledgeable*’.

**Table 1:**
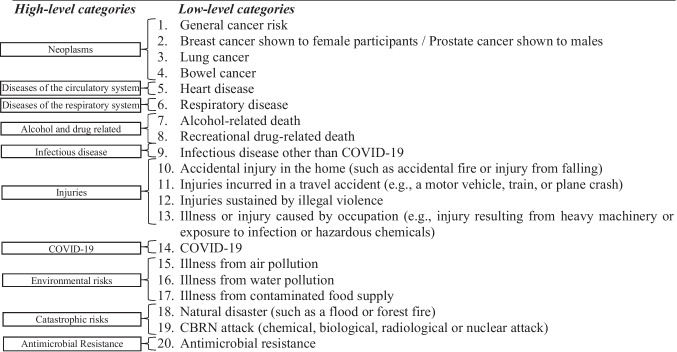
List of causes of death

### Perceived prevalence of avoidable death and accuracy of risk estimations

We measured participants’ perceived prevalence of the ONS’ seven leading causes of avoidable death in the UK (neoplasms, diseases of the circulatory system, diseases of the respiratory system, alcohol and drug-related death, infectious disease, injuries, and ‘other’ risks). Participants were asked, ‘*A significant portion of annual deaths in the UK could be avoided, either through public health intervention or effective treatment. For every 100 avoidable deaths in the UK in 2020, how many do you think, on average, resulted from each of the following causes of avoidable death?*’ Scores were provided on a sliding scale from 0 ‘*none*’ to 100 ‘*all deaths*’. The total summed score for portion of avoidable deaths for all causes had to equal 100. Once the summed total reached 100, subsequent options could not be scored above 0 until previous scores were reduced. All measures presented causes of death in a randomised order to prevent potential scoring bias.

Finally, we calculated the overall accuracy of perceptions of the prevalence of avoidable deaths for each cause. This was calculated by measuring the absolute distance between perceived prevalence scores for each cause and the percentage of avoidable deaths accounted for by each category reported by the ONS’ most recent release on Avoidable Mortality in the UK (Office for National Statistics 2022).

### Analysis

All statistical analyses were performed using R (R Core 2021). The following packages were used for data processing, analysis and visualisation: tidyverse (Wickham et al. 2020), psych (Revelle 2021), Rmisc (Hope 2013), bestNormalize (Peterson 2021), jmv (Selker et al. 2021), factoextra (Kassambara and Mundt 2021), REdaS (Maier 2015), interactions (Long 2020), moments (Komsta and Novomestky 2015), lmtest (Zeileis and Hothorn 2020), olsrr (Hebbali 2020), ggcorrplot (Kassambara 2019), plotly (Sievert 2020) and apaTables (Stanley 2021). The level of statistical significance for all tests was set at *p* < .01 in accordance with guidance for the analysis of medium to large sample sizes (e.g., *n* > 1000), and the need to adjust levels of significance (Priest 2005). For the regression analysis presented, we first ran additional regression analyses to identify significant predictors from our socioeconomic, demographic and occupational exposure variables (age, gender, education, income, neighbourhood safety and occupational risk; see supplement Table S1). Significant predictors were then included as control variables. For violations of the assumption of normality, the R package ‘bestNormalize’ was used to transform variables to produce normally distributed data (Peterson 2021).

The data associated with this study were also used to produce a second article ‘*Individual characteristics associated with perceptions of control over mortality risk and determinants of health effort*’ (Brown et al. 2023). This second article is available as a preprint alongside our data, code, materials and pre-registration with the Open Science Framework [https://osf.io/dgwna].

## Results

Of the 1463 participants included in our analysis, 742 were female, 714 were male and seven reported a different gender identity. Ages ranged from 18 to 87 years (*M* = 45.58, *SD* = 15.53). Years in post-16 education ranged from 0 to 23 years (*M =* 5.12, *SD* = 3.48). Monthly discretionary income ranged from –£1,100 (indicating greater monthly outgoings than incomings) to £7,500 (*M* = 306.31, *SD* = 463.63) and the mean score for subjective discretionary income was 8.50 out of 15 (*SD* = 2.64). Participants indicated that they generally ‘*agree’* that they feel safe in their neighbourhood (*M =* 5.55 out of 7, *SD* = 1.15). Of those in employment (*n* = 999), scores for occupational exposure to the 13 physical risks from the Physical Working Environment Subscale from the European Working Conditions Survey 2015 (Eurofound 2017) ranged from 13–71 out of a possible 91, with the average score (*M =* 23.48, *SD =* 9.47) suggesting that most participants believe they are ‘*almost never*’ exposed to physical risks in the workplace.

### Perceived control over each cause of death

**Fig. 1:**
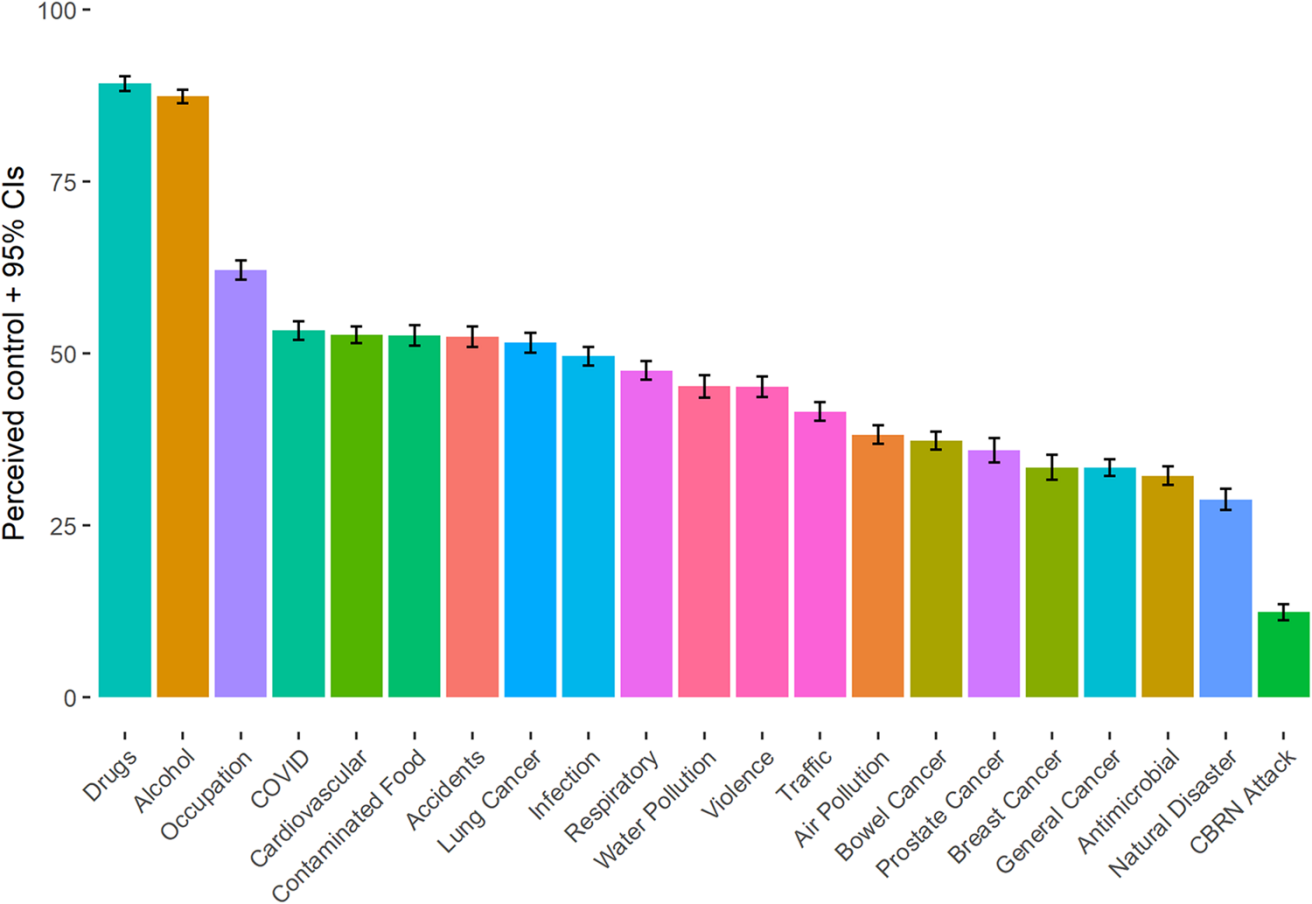
Perceived control over each cause of death. *Bars represent mean scores out of 100 for each cause of death, with 95% confidence intervals. N = 1463 for all causes except breast cancer (n = 742 females) and prostate cancer (n = 714 males)*

The least controllable causes of death were reported to be chemical, biological, radiological or nuclear attacks (*M* = 12.40 out of 100, *SD* = 22.01), natural disasters (*M* = 28.80, *SD* = 29.56) and the threat of antimicrobial resistance (*M* = 32.24, *SD* = 25.92), followed by four of the five categories of cancer risk (*M*_*general*_ = 33.41, *SD*_*general*_ = 24.10; *M*_*breast*_ = 33.45, *SD*_*breast*_ = 25.12; *M*_*prostate*_ = 35.95, *SD*_*prostate*_ = 24.62; *M*_*bowel*_ = 37.38, *SD*_*bowel*_ = 25.39). Risk of death due to drugs and alcohol were considered most controllable (*M*_*drugs*_ = 89.23, *SD*_*drugs*_ = 20.66; *M*_*alcohol*_ = 87.37, *SD*_*alcohol*_ = 19.65; see Fig. 1).

### Perceived likelihood of dying due to each cause of death

**Fig. 2:**
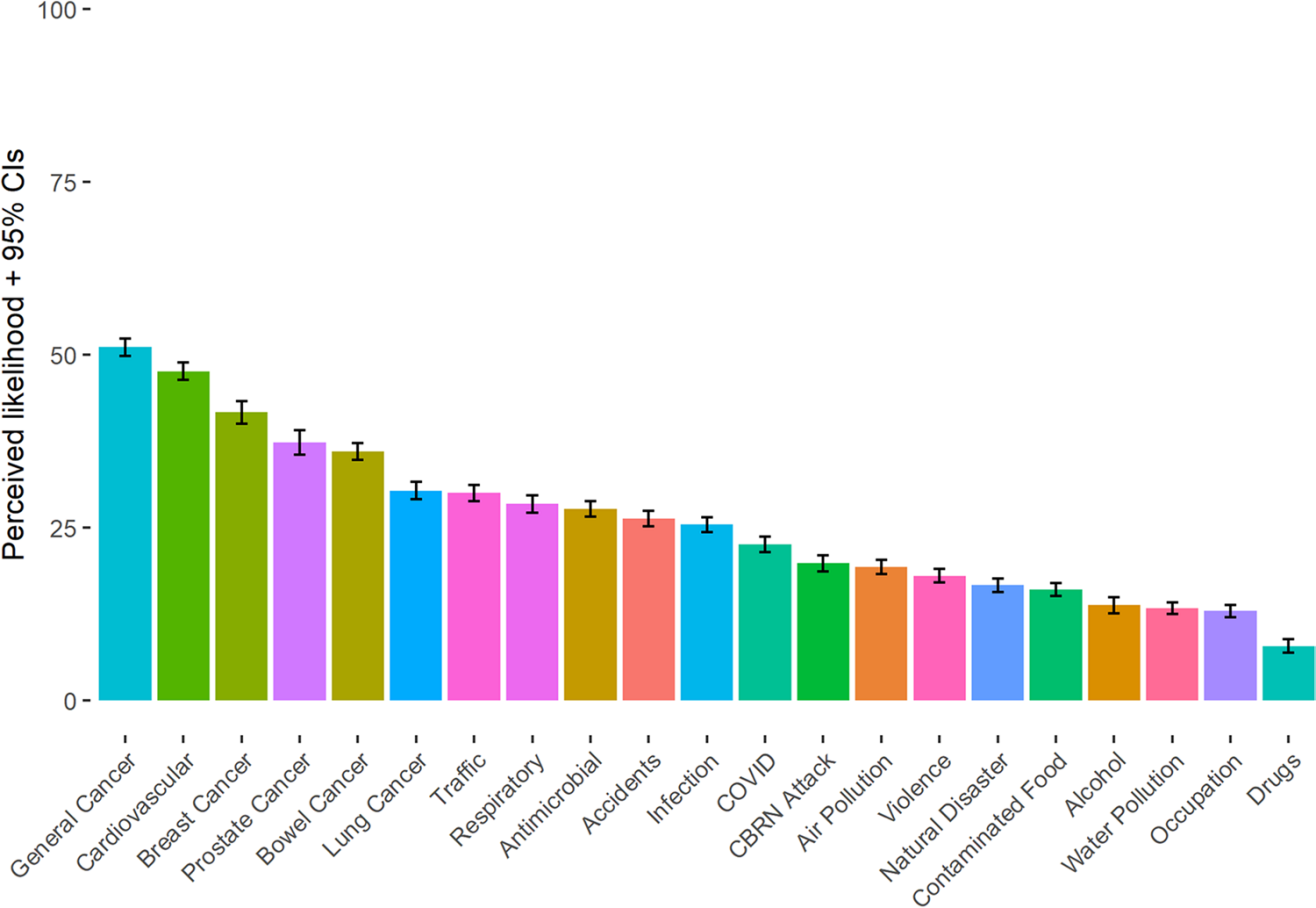
Perceived personal likelihood of dying due to each cause of death. *Bars represent mean scores out of 100 for each cause of death, with 95% confidence intervals. N = 1463 for all causes except breast cancer (n = 742 females) and prostate cancer (n = 714 males)*

Perceived personal likelihood of death was highest for general cancer risk (*M* = 51.11, *SD* = 24.34), followed by cardiovascular risk (*M* = 47.64, *SD* = 24.83), and the remaining four categories of cancer *M*_*breast*_ = 41.70, *SD*_*breast*_ = 22.62; *M*_*prostate*_ = 37.36, *SD*_*prostate*_ = 24.44; *M*_*bowel*_ = 36.03, *SD*_*bowel*_ = 23.48; *M*_*lung*_= 30.38, *SD*_*lung*_ = 24.46. Personal death due to drugs was considered least likely (*M* = 7.91, *SD* = 18.80; see Fig. 2).

**Fig. 3:**
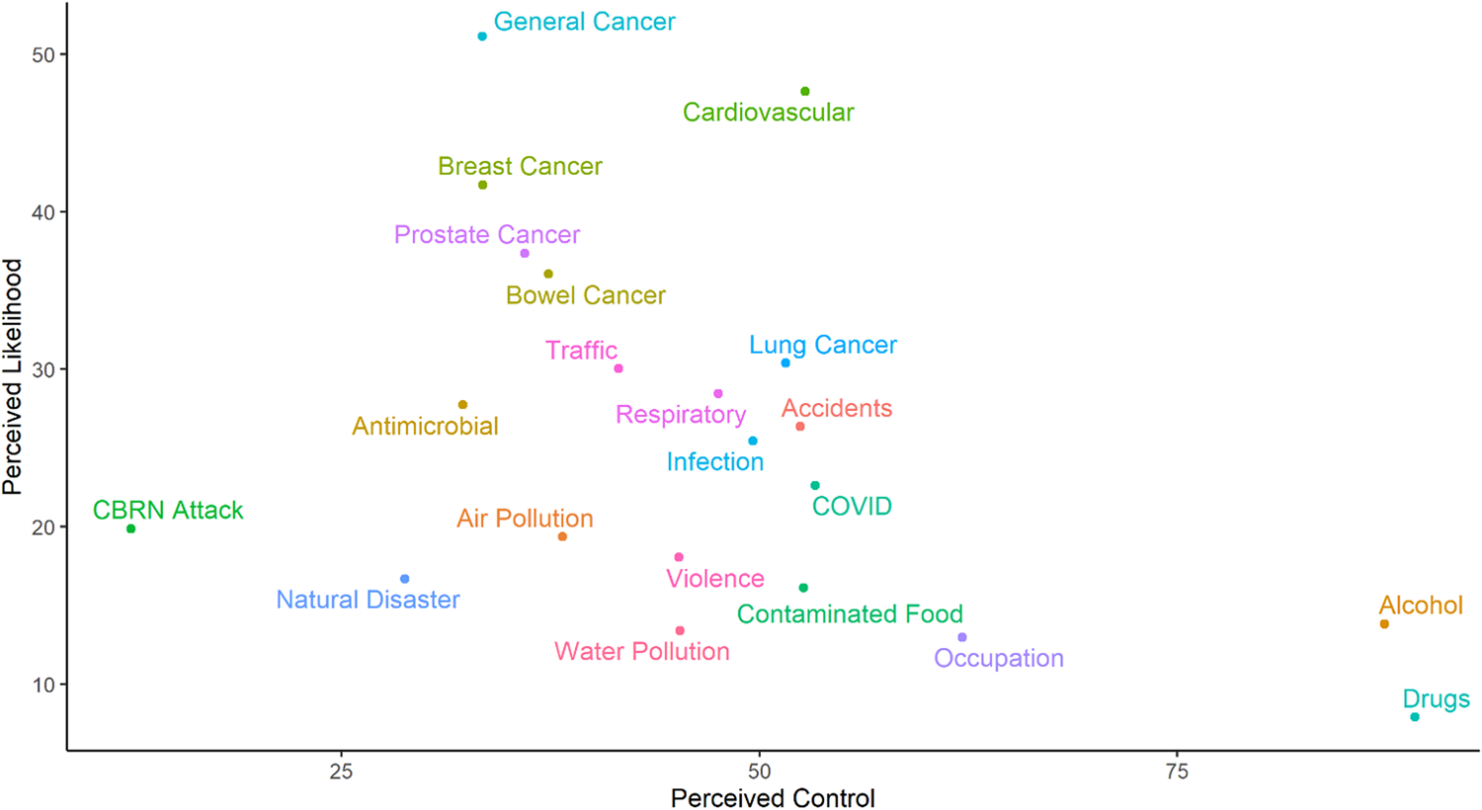
Perceived control and likelihood of death due to each cause of death. *N = 1463 for all causes except breast cancer (n = 742 females) and prostate cancer (n = 714 males).*

Overall, drugs and alcohol were perceived as risks that were both high in control and low in likelihood of death. Cardiovascular disease was considered reasonably controllable and a likely cause of personal death, whereas cancers (with the exception of lung cancer) were considered reasonably uncontrollable and likely causes of personal death (see Fig. 3).

### Perceived uncontrollable mortality risk (PUMR)

The mean score for PUMR was 26.91 out of 100 (*SD* = 17.08). All scores for perceived control over the ten high-level causes of death (see Table 1) were significantly correlated with PUMR, such that greater perceived control over individual risks was associated with lower PUMR. However, the effect sizes were small (ranging from *r* = –.11 to –.29). Furthermore, when included in a regression model containing perceptions of control over individual causes of death as predictors, and PUMR as the outcome variable, only perceived control over circulatory disease (*β* = –.19, 95% CI [–0.25, –0.13]) and perceived control over drugs and alcohol (*β* = –.08, 95% CI [–0.13, –0.02]) were statistically significant predictors of PUMR (see Table 2).

**Table 2:** Regression analyses showing predictors of perceived uncontrollable mortality risk

Predictor	*b*	*b* 95% CI [LL, UL]	*beta*	*beta* 95% CI [LL, UL]	*sr*^*2*^	*sr*^*2*^ 95% CI [LL, UL]	*r*	Fit
(Intercept)	0.00	[–0.05, 0.05]						
Neoplasms	–0.00	[–0.07, 0.06]	–0.00	[–0.06, 0.06]	.00	[–.00, .00]	–.19**	
Circulatory	–0.20**	[–0.26, –0.13]	–0.19	[–0.25, –0.13]	.02	[.01, .04]	–.29**	
Respiratory	–0.03	[–0.09, 0.04]	–0.03	[–0.09, 0.04]	.00	[–.00, .00]	–.22**	
Drugs/alcohol	–0.08**	[–0.14, –0.03]	–0.08	[–0.13, –0.02]	.00	[–.00, .01]	–.15**	
Infections	–0.05	[–0.11, 0.02]	–0.05	[–0.11, 0.02]	.00	[–.00, .00]	–.20**	
Injuries	–0.02	[–0.09, 0.05]	–0.02	[–0.09, 0.05]	.00	[–.00, .00]	–.19**	
COVID19	–0.04	[–0.10, 0.03]	–0.04	[–0.10, 0.02]	.00	[–.00, .00]	–.20**	
Environmental	0.02	[–0.05, 0.09]	0.02	[–0.05, 0.09]	.00	[–.00, .00]	–.17**	
Catastrophic	0.02	[–0.05, 0.08]	0.02	[–0.05, 0.08]	.00	[–.00, .00]	–.11**	
Microbes	–0.06	[–0.11, 0.00]	–0.05	[–0.11, 0.00]	.00	[–.00, .01]	–.15**	
Subjective discretionary income	–0.11**	[–0.16, –0.05]	–0.11	[–0.16, –0.05]	.01	[–.00, .02]	–.19**	
Discretionary income	–0.06*	[–0.11, –0.00]	–0.06	[–0.11, –0.00]	.00	[–.00, .01]	–.15**	
Perceived neighbourhood safety	–0.08**	[–0.13, –0.03]	–0.08	[–0.13, –0.03]	.01	[–.00, .01]	–.14**	
								*R*^*2*^ = .138**
								95% CI [.10,.16]

A significant *b*-weight indicates the beta-weight and semi-partial correlation are also significant. *b *represents unstandardised regression weights. *beta *indicates the standardised regression weights. sr^2^ represents the semi-partial correlation squared. *r *represents the zero-order correlation. LL and UL indicate the lower and upper limits of a confidence interval, respectively. * indicates *p* < .05. ** indicates *p* < .01.

### Certainty of risk estimations and perceived knowledge of causes of death

Perceptions of control and likelihood of death were associated with participants’ certainty of their own risk estimations and their perceived level of knowledge, though the effects were small (see Table 3; (Ellis 2010). The more knowledgeable participants felt they were about a specific cause of death, the more certain they were of their estimation of risk (*r* = .27, 95% CI [.26, .28]). The more certain participants were of their estimation of risk, the more control they felt they had over their risk of death (*r* = .24, 95% CI [.23, .25]). Finally, the more control participants felt they had over their risk of death, the less likely they felt they were to die from a specific cause (*r* = –.18, 95% CI [–.17, –.19]). For full details of the differences between causes of death in perceived certainty and knowledge reported by our sample, see supplement Figs. S1–2.

**Table 3:** Spearman correlations between overall perceptions of control, likelihood, certainty and knowledge of causes of death

Variable	*M*	*SD*	1	2	3
1. Control	47.39	31.54			
2. Likelihood of death	25.17	24.34	–.18**		
			[–.19, –.17]		
3. Certainty of risk estimation	57.39	31.07	.24**	–.14**	
			[.23, .25]	[–.15, –.13]	
4. Personal knowledge	47.56	28.60	.18**	.12**	.27**
			[.17, .19]	[.11, .13]	[.26, .28]

*M* and *SD* are used to represent mean and standard deviation, respectively. Values in square brackets indicate the 95% confidence interval for each correlation. The confidence interval is a plausible range of population correlations that could have caused the sample correlation (Cumming, 2014). * indicates *p *< .05. ** indicates *p* < .01.

### Accuracy of perceived prevalence of avoidable deaths in the UK

**Fig. 4:**
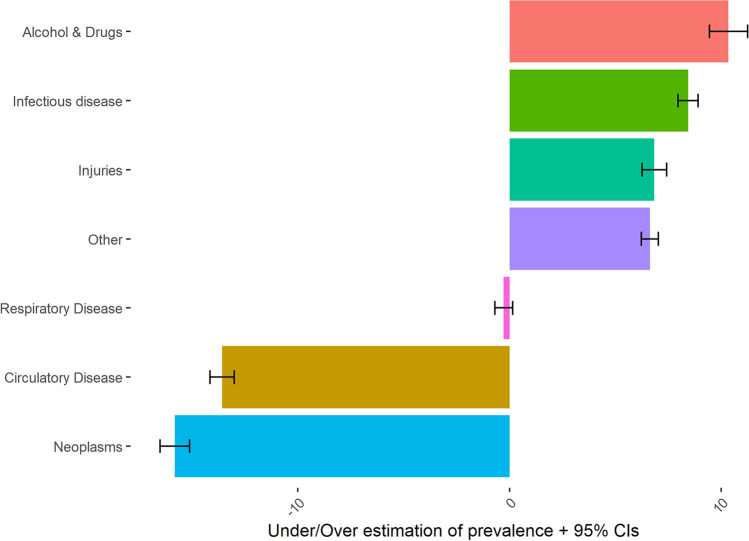
Accuracy of estimation of prevalence of avoidable deaths in the UK

Alcohol and drugs were considered to be the most prevalent category of avoidable death, with participants, on average (mean), estimating that these risks account for 22% of avoidable deaths in the UK. This was followed by cancers which were believed to account for 19% of avoidable deaths. Compared to the actual prevalence of avoidable deaths in the UK (calculated by the ONS’s most recent release on avoidable deaths; Office for National Statistics (2022)), on average, participants overestimated the prevalence of avoidable deaths due to alcohol and drugs by 10%, infectious disease by 8% and injuries by 7%. Participants underestimated the prevalence of avoidable deaths due to cancers by 16% and cardiovascular disease by 14%. Figure 4 shows the accuracy of prevalence estimation for all categories of avoidable death (for a comparison of perceived and actual prevalence of avoidable deaths in the UK, see supplement Fig. S3). For correlational analysis showing the relationships between perceived knowledge of different causes of death and the accuracy of estimations of their prevalence, see supplement Table S2).

**Fig. 5:**
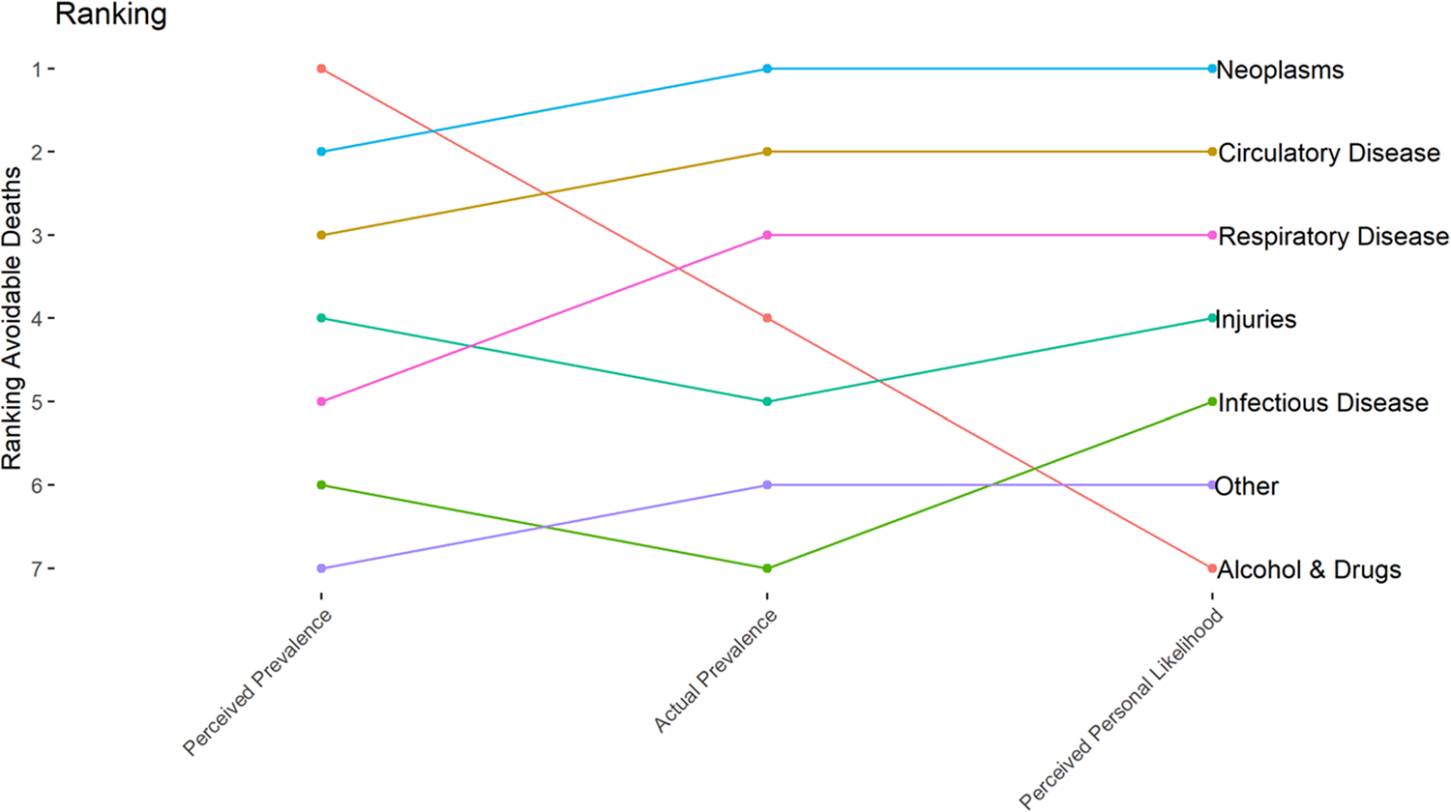
Causes of avoidable death ranked by perceived prevalence in the UK, actual prevalence in the UK, and perceived personal likelihood of death

Finally, causes of avoidable death were ranked in terms of participants’ perceived level of prevalence, the actual prevalence of avoidable death in the UK, and participants’ perceived personal likelihood of death. The top three causes of avoidable death that participants reported they were most likely to die from corresponded with the three most prevalent causes of avoidable death in the UK (cancer, circulatory disease and respiratory disease). Conversely, drugs and alcohol were believed to be the most prevalent causes of avoidable death in the UK, but are ranked fourth in actual prevalence, and were ranked last in perceived personal likelihood of death (see Fig. 5).

## Discussion

This study investigated perceptions of control over different causes of death and aimed to capture the accuracy of risk estimations by measuring perceived prevalence of avoidable deaths in the UK. Our findings reflect the risk perceptions and control beliefs of an online UK-representative sample recruited in May 2022. By considering the influence of specific control beliefs on overall levels of PUMR, as well as the differences in perceptions observed between causes of death, we provide insights that may inform interventions aimed at encouraging preventative health behaviours.

### Perceptions of control and likelihood for different causes of death

Perceptions of control and likelihood of death varied by cause but largely reflected findings from past literature, actual rates of prevalence in the UK, as well as expected levels of control over certain risks. For example, drugs and alcohol were considered the most controllable causes of death. Notwithstanding the neurobiological, genetic and environmental factors that can influence addiction and resulting death (Koob and Volkow 2016; Wong et al. 2011), levels of consumption of alcohol and illicit drugs are often associated with self-efficacy and individual control (West and Brown 2013). Therefore, it is unsurprising that participants typically reported feeling that they can control their risk of dying due to drugs and alcohol but cannot control the risks they face from natural disasters or chemical, biological, radiological or nuclear attacks. Similarly, the present study offers support for previous findings that people typically feel they have moderate control over their risk of dying due to cardiovascular disease but perceive little control over their risk of cancer-related death (Wang et al. 2009; Brown et al. 2022; Curtin et al. 2018). Finally, participants rated cancer and cardiovascular disease as the most probable causes of their own death. This mirrors the high level of prevalence of these causes of death in the UK, as they were the two most reported categories of avoidable death in 2020 (Office for National Statistics 2022).

By considering perceptions of control and likelihood together, cancer-related death (due to either bowel, breast, prostate or general cancer risk) was perceived by our sample as both likely to occur, and largely beyond individual control. All five responses for cancer scored in the top six most likely causes of individual death, and only natural disasters, catastrophic attacks and antimicrobial resistance were considered less controllable. The exception being lung cancer, which was considered less likely and more controllable than other types of cancer. This is likely due to the success of extensive health campaigns to inform the public of the harms of smoking (Durkin et al. 2012; Proctor 2012). Feeling more knowledgeable about a cause of death was associated with greater certainty over risk estimations which in turn was associated with higher perceived control. Our findings suggest that people believe they have a moderate degree of knowledge concerning their risk of dying from cancer but are unsure as to the accuracy of their risk estimations. The combination of high perceived likelihood and low perceived control suggests that cancer may be a suitable target for interventions aimed at increasing perceptions of control to encourage preventative health behaviours. As discussed, the UMRH stresses that those who believe they are more likely to die as a result of factors that are beyond their control should be less motivated to engage in positive health behaviours (Pepper and Nettle 2014a). Furthermore, findings from the broader fear appeal literature (including work on protection motivation theory and extended parallel process model) highlight that where individuals consider a risk to be largely uncontrollable, health messages that increase levels of fear by highlighting the likelihood of a threat are expected to be ineffective in encouraging healthy behaviours (Witte 1992; Popova 2012; Witte and Allen 2000; Rogers 1975; Rogers et al. 1983; Floyd et al. 2000; Bui et al. 2013). Increasing cancer rates in recent years have been widely reported in the UK, with the estimated lifetime risk of being diagnosed with cancer now 1 in 2 (Cancer Research UK 2018a). This widely reported increase in cancer rates is likely to increase levels of fear associated with the threat of cancer. In response to this increasing threat, public health narratives have focussed on the importance of highlighting timely screening and treatment. For example, focus groups with NHS health professionals reported that health communicators typically highlight the importance of screening for cancer risk, whereas the influence of lifestyle on cancer risk is rarely discussed (Usher-Smith et al. 2017). However, doing more to emphasise the lifestyle and behavioural changes that individuals can make to lessen their risk of developing cancer may help to address low levels of perceived control and ultimately to encourage preventative health behaviours.

Identifying and communicating the causal connections between certain behaviours and the incidence of specific types of cancer is complex and difficult to achieve (Blackadar 2016). Given the complexity of the relationships between health behaviours and specific types of cancer, a recent review of diet, nutrition and cancer risk suggested that public health communicators should aim to highlight the importance of positive health behaviours for the reduction of general cancer risk, rather than targeting specific types of cancer (Key et al. 2020). From our cancer variables, the general risk of a cancer-related death was typically rated as more likely and less controllable than any of the specific types of cancer. Therefore, the finding that people in the UK typically perceived general cancer risk as being highly likely and largely beyond their personal control may further suggest that it is a suitable target for interventions aimed at improving perceptions of control to encourage preventative health behaviours.

### The relationship between perceptions of control over specific causes of death and PUMR

Contrary to the predictions in our preregistration, perceptions of control for eight out of ten high-level causes of death were found not to be statistically significant predictors of overall levels of PUMR. Only perceptions of control over cardiovascular disease (small effect size; (Ellis 2010) and drugs and alcohol (trivial effect size; (Ellis 2010) predicted PUMR. This suggests that overall perceptions of control over mortality risk are not largely driven by perceptions of control over specific causes of death. We discuss a number of possible explanations for why this may be the case.

The UMRH suggests that humans may have developed an adaptive psychological mechanism that responds to environmental cues of risk to determine the optimal level of investment in preventative health (Pepper and Nettle 2014a, c, 2017a). Previous research has shown how humans evolved to respond to immediate threats from surrounding predators (Blanchard et al. 2011) and to detect indicators of imminent violence (White et al. 2012). It has also been suggested that humans evolved to respond to cues of unpredictability in their environment, such as the loss of a parent or changes to one’s surroundings (Young et al. 2020). However, given the relative safety and stability of the modern world compared to our ancestral environment, it is unclear how equipped we are to detect and respond appropriately to the most likely causes of harm we face today. For example, as previously discussed, cancer is the most prevalent category of avoidable death in the UK (Office for National Statistics 2022) and the causal pathways between lifestyle behaviours and cancer-related death are complex and difficult to establish (Blackadar 2016). Given this complexity, and the often lengthy timelines of cancer development (Cancer Research UK 2018b), we are unlikely to have evolved psychological mechanisms capable of recognising cues of increasing cancer rates and the prevalence of lifestyle behaviours that are harmful to our health. Similarly, with respect to wider existential threats, though humans are able to detect the short-term variability of local weather (Mumenthaler et al. 2021), it is unlikely that we have evolved to detect long-term patterns of climate change. Furthermore, the channels of information through which we learn about the risks most likely to cause us harm are sure to differ from our ancestral environments. For example, increasing numbers of people are using social media and internet search engines to seek out information about risks to their health and mortality (Liang and Wang 2021). It is possible that individual assessments of control over specific causes of death may be more influenced by informational environments than physical environmental conditions. Therefore, the safety and stability we experience compared to our ancestral environments, the difficulty of personally detecting the complex patterns of the mortality risks we face today, and the informational environments through which we learn about specific risks, may all help to explain why perceptions of control over common contemporaneous causes of death do not appear to drive overall perceptions of uncontrollable mortality risk. Given the difficulty of establishing the drivers of PUMR, future studies may look to use machine learning models to unravel the ties between informational environments and subsequent risk perceptions. For example, Aka and Bhatia (2022) recently developed a language model using text explanations on NHS websites to quantitatively predict lay health perceptions of the severity of everyday diseases. Further study may also look to compare objective measures of environmental risk with informational environments to investigate the influence they have on perceptions of specific risk.

If overall levels of PUMR are not largely driven by aggregating perceived control over specific causes of death, what factors do shape PUMR? It is possible that upbringing, experiences in adolescence, and cultural narratives play a role in determining levels of PUMR. For example, people who experience childhood trauma typically report greater external locus of control than those who do not (Roazzi et al. 2016), experiences of violence during adolescence predict heightened perceptions of risk in adulthood (Macmillan 2000), and different cultural backgrounds predict optimism and overall perceived risk with respect to external threats, such as natural disasters and terrorist attacks (Gierlach et al. 2010). Additionally, researchers in the UK have found that patients with South Asian heritage are more likely to believe that heart disease is unpreventable and that it is caused by fate (Curtin et al. 2018; Darr et al. 2008). Understanding the influence of cultural narratives around heart disease is important as they may impact the willingness of patients to engage in preventative health behaviours. In the present study, perceived control over cardiovascular disease was found to be the strongest predictor of overall levels of perceived uncontrollable mortality risk. Given the discussed negative association between PUMR and reported health effort, understanding beliefs about the controllability of cardiovascular disease may help to encourage positive health behaviours. Only drugs and alcohol, occupational and COVID-related deaths were considered more controllable by our sample. Despite indicating a reasonable degree of perceived control, our sample considered cardiovascular disease to be the second most likely cause of personal death after cancer. This high degree of perceived likelihood may indicate that, although participants felt their risk of a cardiovascular-related death could potentially be controlled, they are not currently acting within their powers to mitigate the risk. Barriers to pursuing a healthy lifestyle can include poor mental health as well as experiences of stigma (structural, social and self) (Graham et al. 2013). Research suggests that health and wellbeing related services are key to providing a structural element through which society can promote healthier living, in cooperation with the individual (Tuohimaa 2014). For some people, a strong sensory attraction to unhealthy palatable foods, and to overeating, may also negatively impact the pursuit of a healthy lifestyle (Carlos et al. 2014). Researchers have proposed health interventions that aim to communicate the sensory pleasure, rather than long-term health benefits, of a healthy lifestyle. For example, taste-focused labels have been found to increase healthy food choices when compared to health-focussed labels in various real-world settings (Turnwald and Crum 2019). There is also an established body of empirical research on neighbourhood effects on health behaviours (Galster 2012). The topography of an area, and whether residential areas are within walking distance of essential services is likely to influence aerobic activity (Flowerdew et al. 2008). Additionally, living in a food desert creates a clear barrier to pursuing a healthy lifestyle. ‘Food deserts’ refer to neighbourhoods that have limited access to nutritious and affordable food (Whitacre et al. 2009). Similarly, living in close proximity to processed food outlets poses additional challenges to maintaining a healthy diet. Further study may look to disentangle the various neighbourhood effects that can influence an individual’s motivation and ability to mitigate their risk of a cardiovascular-related death.

### Perceived prevalence of avoidable death

The findings from the present study offer support for the prediction made in our preregistration concerning the presence of a *primary bias* in risk perception, whereby people typically overestimate rare risks to their health and underestimate common risks (Hakes and Viscusi 2004; Slovic 1978; Lichtenstein et al. 1978). We found that more common causes of avoidable death (cancers and cardiovascular disease) were underestimated when compared to the actual prevalence in the UK, whereas less common causes of avoidable death (injuries, infectious disease, drugs and alcohol) were overestimated. A possible explanation for this bias in the estimated prevalence of different categories of avoidable death is the way in which the media report on mortality risks. For example, Ritchie and Roser (2018) found that in the US, despite around one-third of deaths being due to heart disease, this cause of death receives only 2–3% of media coverage relating to death. They also found that though just under one-third of US deaths resulted from cancer, this mortality risk receives only 13–14% of media coverage. Contrast this with media coverage of violent deaths; which represents more than two-thirds of overall coverage on causes of death, despite accounting for fewer than 3% of annual US deaths. Additionally, Pilar et al. (2020) recently highlighted how the actual causes of death in the US are misaligned with health-related media attention, as well as with policy attention and federal spending. They suggested that this misalignment between media coverage and the actual prevalence of different causes of death is likely to shape the public’s perception of the health risks they face.

Media coverage may be particularly impactful on perceptions of risk concerning drugs and alcohol. For example, media sensationalism and alarmist rhetoric in response to high-profile overdoses and celebrity deaths can strongly affect perceptions of risk (Orsini 2015; Murji 2020; Brown and Midberry 2022). In the UK, there has been a growing focus on binge drinking and anti-social behaviour by the media, often framing problem drinking as a criminal justice issue rather than as a health issue (Meier 2010). Extreme portrayals of alcohol and drug-related issues by the media may affect perceptions of control and the perceived likelihood of a substance-related death. Our findings suggest that people in the UK think that the risks from drugs and alcohol are highly controllable and unlikely to result in their own death. Our sample reported feeling knowledgeable about these risks and moderately certain in their estimations. Despite this, our sample substantially overestimated the prevalence of drug and alcohol-related deaths in the UK. This might suggest that drug and alcohol-related deaths are believed to be risks that are likely to be experienced by others, but not by oneself. An exploration of UK drinking trends by the Joseph Rowntree foundation found that people typically underestimate the amount of alcohol they consume (Smith and Foxcroft 2009). Furthermore, in a cross-sectional study of the drinking habits of approximately 10,000 global participants, the tendency to underestimate one’s own alcohol consumption was found to be common in the US, Canada and Australia, and most common among UK males (Garnett et al. 2015). This combination of sensationalising drug and alcohol-related issues, but underestimating personal consumption, may decrease the perceived likelihood of suffering drug or alcohol-related deaths, whilst increasing the perceived prevalence of risk in the wider community. This suggests a growing need for ethical journalism when reporting on issues that are likely to influence public perceptions of risk and, thereby, subsequent health behaviours. For example, research during the COVID-19 pandemic highlighted the importance of consulting public health professionals when communicating health threats to the public (Kyriakidou et al. 2021).

## Limitations

The results of this study are not without limitation. For example, it is possible that the causes of death that we included in our survey do not fully represent those with the greatest influence on perceptions. We included a broad range of causes of death by referencing the ONS’ categories of avoidable death in the UK, the UK National Risk Register 2020, and recent qualitative findings on perceptions of control over risk (Cabinet Office 2020; Office for National Statistics 2022; Brown et al. 2022). However, beliefs surrounding other causes of death that are salient to the public may have influenced reported perceptions of risk. For example, of all deaths registered in 2019 in England and Wales, 13% were due to dementia and Alzheimer’s disease (Office for National Statistics 2020). Despite the increasing prevalence of this cause of death in the UK, it is not classed as an avoidable death. Given our focus on the relationships between perceptions of risk, health behaviours and the reduction of avoidable deaths, we chose not to include this cause of death in our survey. Future research may look to include a broader range of causes of death when studying public perceptions, as well as to investigate public knowledge concerning which health risks are or are not personally avoidable.

## Conclusion

Our findings suggest that people perceive cancer-related deaths to be both likely to happen to them, and largely beyond their control. This has implications for preventative health behaviours, as the UMRH suggests that those who believe they are more likely to die as a result of factors that are beyond their control should be less motivated to engage in positive health behaviours. Ironically, reduced motivation to behave healthfully may increase risk of cancer, thus perpetuating a feedback loop. We suggest that more can be done by public health communicators to emphasise the lifestyle and behavioural changes that individuals can make to reduce their general cancer risk.

Perceptions of control over specific causes of death were found not to be strong predictors of overall levels of perceived uncontrollable mortality risk. We recommend that future studies investigate the roles that developmental, cultural and informational environments play in driving perceptions of control over risk. The exception to this trend was perceptions of control over cardiovascular disease, which was negatively associated with overall PUMR. Risk of death due to cardiovascular disease was considered controllable, relative to other risks, yet was still rated the second most likely cause of individual death. We suggest that further research is needed to study and unravel the numerous neighbourhood effects that may discourage people from maintaining a healthy heart.

Finally, our findings offer support for the *primary bias* in risk perceptions, by investigating a previously unexplored UK population with respect to the prevalence of avoidable death. The portion of UK avoidable deaths due to drugs and alcohol was substantially overestimated by our sample, despite being considered the least likely cause of personal death. Health professionals should work to counteract the tendency to underestimate alcohol consumption and we call for greater journalistic responsibility when reporting health risks to the public.

## Supplementary information

ESM 1 (DOCX 142 kb)

## Data Availability

All data will be made available upon publication.

## Code availability

All code (R scripts) will be made available upon publication.

## References

[CR1] Aka A, Bhatia S (2022). Machine learning models for predicting, understanding, and influencing health perception. J Assoc Consum Res.

[CR2] An R, Yang Y, Hoschke A, Xue H, Wang Y (2017). Influence of neighbourhood safety on childhood obesity: a systematic review and meta-analysis of longitudinal studies. Obes Rev.

[CR3] Blackadar CB (2016). Historical review of the causes of cancer. World J Clin Oncol.

[CR4] Blanchard DC, Griebel G, Pobbe R, Blanchard RJ (2011). Risk assessment as an evolved threat detection and analysis process. Neurosci Biobehav Rev.

[CR5] Brown DK, Midberry J (2022). Social media news production, emotional Facebook reactions, and the politicization of drug addiction. Health Commun.

[CR6] Brown R, Pepper G (2022) Improving the safety, quality, and stability of people's environments should encourage healthy behaviour. Psychologist 35:64–67

[CR7] Brown RD, Pepper G (2023) The Uncontrollable Mortality Risk Hypothesis of Health Behaviour: a position paper. PREPRINT. 10.31219/osf.io/py7dw

[CR8] Brown R, Coventry L, Pepper G (2021a) COVID-19: the relationship between perceptions of risk and behaviours during lockdown. J Public Health 1–11. 10.1007/s10389-021-01543-9 10.1007/s10389-021-01543-9 PMC8118375 34007783

[CR9] Brown R, Coventry L, Pepper G (2021). Information seeking, personal experiences, and their association with COVID-19 risk perceptions: demographic and occupational inequalities. J Risk Res.

[CR10] Brown R, Sillence E, Pepper G (2022). A qualitative study of perceptions of control over potential causes of death and the sources of information that inform perceptions of risk. Health Psychol Behav Med.

[CR11] Brown R, Sillence E, Pepper G (2023) Individual characteristics associated with perceptions of control over mortality risk and determinants of health effort. PREPRINT. 10.31219/osf.io/dpgvf 10.1111/risa.14243 37871995

[CR12] Bui L, Mullan B, McCaffery K (2013). Protection motivation theory and physical activity in the general population: A systematic literature review. Psychol Health Med.

[CR13] Cabinet Office (2020) National Risk Register 2020. UK Government

[CR14] Cancer Research UK (2018a) Lifetime risk of cancer. https://www.cancerresearchuk.org/health-professional/cancer-statistics/risk/lifetime-risk#heading-Zero

[CR15] Cancer Research UK (2018b) Science Surgery: ‘How quickly do tumours develop?’

[CR16] Carlos S, de Irala J, Hanley M, Martínez-González MÁ (2014). The use of expensive technologies instead of simple, sound and effective lifestyle interventions: a perpetual delusion. J Epidemiol Community Health.

[CR17] Clerk AM (2021). Beware of neglect of non-COVID patients in COVID era. Indian Crit Care Med: Peer-reviewed, Off Publ Indian Soc Crit Care Med.

[CR18] Core R (2021). Core R: a language and environment for statistical computing.

[CR19] Curtin KD, Berry TR, Courneya KS, McGannon KR, Norris CM, Rodgers WM, Spence JC (2018). Investigating relationships between ancestry, lifestyle behaviors and perceptions of heart disease and breast cancer among Canadian women with British and with South Asian ancestry. Eur J Cardiovasc Nurs.

[CR20] Darr A, Astin F, Atkin K (2008). Causal attributions, lifestyle change, and coronary heart disease: illness beliefs of patients of South Asian and European origin living in the United Kingdom. Heart Lung.

[CR21] Drews S, Savin I, Van Den Bergh JC, Villamayor-Tomás S (2022). Climate concern and policy acceptance before and after COVID-19. Ecol Econ.

[CR22] Durkin S, Brennan E, Wakefield M (2012). Mass media campaigns to promote smoking cessation among adults: an integrative review. Tob Control.

[CR23] Ellis PD (2010) The essential guide to effect sizes: Statistical power, meta-analysis, and the interpretation of research results. Cambridge university press

[CR24] Eurofound (2017) Sixth European Working Conditions Survey – Overview report (2017 update). Publications Office of the European Union. Luxembourg

[CR25] Flowerdew R, Manley DJ, Sabel CE (2008). Neighbourhood effects on health: does it matter where you draw the boundaries?. Soc Sci Med.

[CR26] Floyd DL, Prentice-Dunn S, Rogers RW (2000). A meta-analysis of research on protection motivation theory. J Appl Soc Psychol.

[CR27] Galster GC (2012) The mechanism (s) of neighbourhood effects: theory, evidence, and policy implications. In: Neighbourhood effects research: New perspectives. Springer, pp 23-56

[CR28] Garnett C, Crane D, West R, Michie S, Brown J, Winstock A (2015). Normative misperceptions about alcohol use in the general population of drinkers: a cross-sectional survey. Addict Behav.

[CR29] Gierlach E, Belsher BE, Beutler LE (2010). Cross-cultural differences in risk perceptions of disasters. Risk Anal.

[CR30] Graham C, Griffiths B, Tillotson S, Rollings C (2013). Healthy living? By whose standards? Engaging mental health service recipients to understand their perspectives of, and barriers to, healthy living. Psychiatric Rehabilit J.

[CR31] Hakes JK, Viscusi WK (2004). Dead reckoning: Demographic determinants of the accuracy of mortality risk perceptions. Risk Anal.

[CR32] Hebbali A (2020) olsrr: Tools for building OLS regression models. R package version 0(5):3

[CR33] Hertzog MA (2008). Considerations in determining sample size for pilot studies. Res Nurs Health.

[CR34] Holakouie-Naieni K, Nematollahi S (2020). Re: Neglected major causes of death much deadlier than COVID-19. J Res Health Sci.

[CR35] Hope R (2013). Rmisc: Rmisc: Ryan Miscellaneous. R package version.

[CR36] Kassambara A (2019). ggcorrplot: visualization of a correlation matrix using 'ggplot2'. R package version.

[CR37] Kassambara A, Mundt F (2021) Factoextra: extract and visualize the results of multivariate data analyses; R package version 1.0. 7. 2020

[CR38] Key TJ, Bradbury KE, Perez-Cornago A, Sinha R, Tsilidis KK, Tsugane S (2020) Diet, nutrition, and cancer risk: what do we know and what is the way forward? BMJ 368 10.1136/bmj.m511 PMC7190379 32139373

[CR39] Komsta L, Novomestky F (2015) moments: Moments, cumulants, skewness, kurtosis and related tests. R package version 0.14

[CR40] Koob GF, Volkow ND (2016). Neurobiology of addiction: a neurocircuitry analysis. Lancet Psychiatry.

[CR41] Kyriakidou M, Morani M, Soo N, Cushion S (2021) Reporting from the front line: the role of health workers in UK television news reporting of COVID-19. In: Communicating COVID-19. Springer, pp 41-58

[CR42] Liang B, Wang Y (2021). Conceptualizing an ecological model of Google search and Twitter data in public health. Empowering human dynamics research with social media and geospatial data analytics.

[CR43] Lichtenstein S, Slovic P, Fischhoff B, Layman M, Combs B (1978). Judged frequency of lethal events. J Exp Psychol Hum Learn Mem.

[CR44] Long J (2020) interactions: Comprehensive, user-friendly toolkit for probing interactions (1.1. 3)[Computer software]

[CR45] Macmillan IR (2000) Growing up scared, the effects of violent victimization in adolescence on adult socio-economic attainment

[CR46] Maier M (2015) `R: Einführung durch angewandte Statistik”_. R package version 0.9.3

[CR47] Meier PS (2010). Polarized drinking patterns and alcohol deregulation: trends in alcohol consumption, harms and policy: United Kingdom 1990–2010. Nordic Stud Alcohol Drugs.

[CR48] Mumenthaler C, Renaud O, Gava R, Brosch T (2021). The impact of local temperature volatility on attention to climate change: evidence from Spanish tweets. Glob Environ Chang.

[CR49] Murji K (2020). The agony and the ecstasy: drugs, media and morality. The control of drugs and drug users.

[CR50] Nettle D (2010). Why are there social gradients in preventative health behavior? A perspective from behavioral ecology. PLoS One.

[CR51] Office for National Statistics (2020) Dementia and Alzheimer's disease deaths including comorbidities, England and Wales: 2019 registrations

[CR52] Office for National Statistics (2021) Data collection changes due to the pandemic and their impact on estimating personal well-being. People, population and community

[CR53] Office for National Statistics (2022) Avoidable mortality in Great Britain: 2020. vol Health and social care

[CR54] O'Guinn TC, Wells WD (1989) Subjective Discretionary Income. Marketing Research 1 (1)

[CR55] Orsini MM (2015) Media narratives and drug prohibition: a content analysis of themes and strategies promoted in network news coverage, 2000-2013

[CR56] Pepper GV, Nettle D (2014). Out of control mortality matters: the effect of perceived uncontrollable mortality risk on a health-related decision. PeerJ.

[CR57] Pepper GV, Nettle D (2014). Perceived extrinsic mortality risk and reported effort in looking after health. Hum Nat.

[CR58] Pepper GV, Nettle D (2014c) Socioeconomic disparities in health behaviour: an evolutionary perspective. In: Applied evolutionary anthropology. Springer, pp 225-243

[CR59] Pepper GV, Nettle D (2017). The behavioural constellation of deprivation: Causes and consequences. Behav Brain Sci.

[CR60] Pepper GV, Nettle D (2017). Strengths, altered investment, risk management, and other elaborations on the behavioural constellation of deprivation. Behav Brain Sci.

[CR61] Peterson R (2021). Finding optimal normalizing transformations via bestNormalize. R J.

[CR62] Peterson CH, Peterson NA, Powell KG (2017). Cognitive interviewing for item development: validity evidence based on content and response processes. Meas Eval Couns Dev.

[CR63] Pilar MR, Eyler AA, Moreland-Russell S, Brownson RC (2020). Actual causes of death in relation to media, policy, and funding attention: examining public health priorities. Front Public Health.

[CR64] Poorolajal J (2020). Neglected major causes of death much deadlier than COVID-19. J Res Health Sci.

[CR65] Popova L (2012). The extended parallel process model: illuminating the gaps in research. Health Educ Behav.

[CR66] Priest R (2005) Statistical/substantive, interpretations and data limitations. In Encyclopedia of social measurement. Elsevier, pp. 671–674

[CR67] Prins RG, Kamphuis CBM, van Empelen P, Beenackers MA, Brug J, Mackenbach JP, Oenema A (2013) Explaining socio-demographic differences in disengagement from sports in adolescence. Eur J Pub Health 23(5):811–816. 10.1093/eurpub/cks188 10.1093/eurpub/cks188 23302764

[CR68] Proctor RN (2012). The history of the discovery of the cigarette–lung cancer link: evidentiary traditions, corporate denial, global toll. Tob Control.

[CR69] Rader C, Comish R, Burckel D (2011) The effectiveness of single vs multiple-item measures of subjective discretionary income in predicting family purchasing behavior In: Proceedings of ASBBS Las Vegas. vol 1

[CR70] Revelle W (2021). psych: Procedures for Personality and Psychological Research (Version R package version 2.0. 12).

[CR71] Ritchie H, Roser M (2018) Causes of death. Our World in Data

[CR72] Roazzi A, Attili G, Pentima LD, Toni A (2016) Locus of control in maltreated children: the impact of attachment and cumulative trauma. Psicologia: Reflexão e Crítica 29

[CR73] Rogers RW (1975). A protection motivation theory of fear appeals and attitude change1. J Psychol.

[CR74] Rogers R, Cacioppo J, Petty R (1983). Social psychophysiology: a sourcebook.

[CR75] Selker R, Love J, Dropmann D, Moreno V (2021) jmv: The 'jamovi' Analyses. R package version 2.0

[CR76] Sievert C (2020) Chapman and Hall/CRC; 2020. Interactive Web-Based Data Visualization with R, plotly, and shiny

[CR77] Slovic P (1978) The psychology of protective behavior

[CR78] Smith L, Foxcroft D (2009). Drinking in the UK. An exploration of trends.

[CR79] Stanley D (2021) apaTables: create American Psychological Association (APA) style tables. R package version 2.0.8

[CR80] Tuohimaa H (2014). In search of an empowering and motivating personal wellbeing pathway for Finnish heart patients. SpringerPlus.

[CR81] Turnwald BP, Crum AJ (2019). Smart food policy for healthy food labeling: Leading with taste, not healthiness, to shift consumption and enjoyment of healthy foods. Prev Med.

[CR82] United Nations (2022) The UN and the war in Ukraine: key information. Everything you need to know about the UN response to the war in Ukraine

[CR83] Usher-Smith JA, Silarova B, Ward A, Youell J, Muir KR, Campbell J, Warcaba J (2017). Incorporating cancer risk information into general practice: a qualitative study using focus groups with health professionals. Br J Gen Pract.

[CR84] Wang C, O'Neill SM, Rothrock N, Gramling R, Sen A, Acheson LS, Rubinstein WS, Nease DE, Ruffin MT (2009). Comparison of risk perceptions and beliefs across common chronic diseases. Prev Med.

[CR85] West R, Brown J (2013) Theory of addiction. Second edn. Wiley Blackwell. 10.1002/9781118484890.ch5

[CR86] Whitacre PT, Tsai P, Mulligan J (2009) The public health effects of food deserts. In: Workshop Summary 25032337

[CR87] White AE, Kenrick DT, Li YJ, Mortensen CR, Neuberg SL, Cohen AB (2012). When nasty breeds nice: Threats of violence amplify agreeableness at national, individual, and situational levels. J Pers Soc Psychol.

[CR88] Wickham H, Averick M, Bryan J, Chang W, McGowan L, François R, Grolemund G, Hayes A, Henry L, Hester J (2020). Welcome to the Tidyverse. J Open Source Softw.

[CR89] Witte K (1992). Putting the fear back into fear appeals: the extended parallel process model. Commun Monogr.

[CR90] Witte K, Allen M (2000). A meta-analysis of fear appeals: implications for effective public health campaigns. Health Educ Behav.

[CR91] Wong CC, Mill J, Fernandes C (2011). Drugs and addiction: an introduction to epigenetics. Addiction.

[CR92] YouGov (2021) Research Q&A's. yougov.co.uk/about/panel-methodology/research-qs/

[CR93] Young ES, Frankenhuis WE, Ellis BJ (2020). Theory and measurement of environmental unpredictability. Evol Hum Behav.

[CR94] Zeileis A, Hothorn T (2020). Diagnostic checking in regression relationships. R News.

