# Supplementary material for: Perceptions of control over different causes of death and the accuracy of risk estimations
Source: Z Gesundh Wiss. 2023 Apr 14:1–14. Online ahead of print. doi: 10.1007/s10389-023-01910-8 (PMC10102679; doi:10.1007/s10389-023-01910-8)
Supplement: Supplementary file 1 — (DOCX 142 kb) [file 10389_2023_1910_MOESM1_ESM.docx]

**Perceptions of control over different causes of death**

**and the accuracy of risk estimations**

**Supplement**


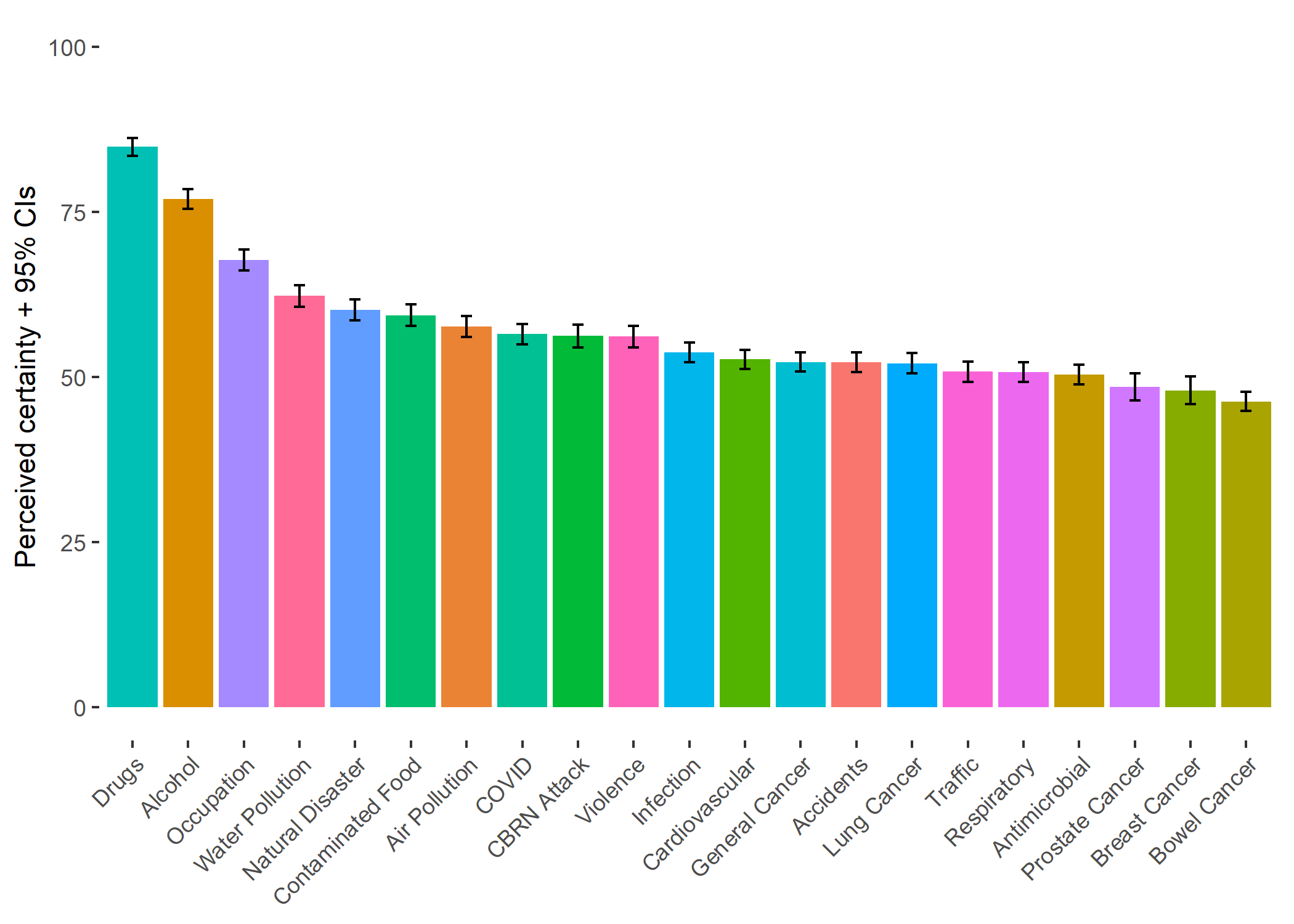


**Figure S1.** Perceived certainty of risk estimation for each cause of death.

*^Bars represent mean scores out of 100 for each cause of death, with 95% confidence intervals. N = 1,463 for all causes except breast cancer (n = 742 females) and prostate cancer (n = 714 males).^*


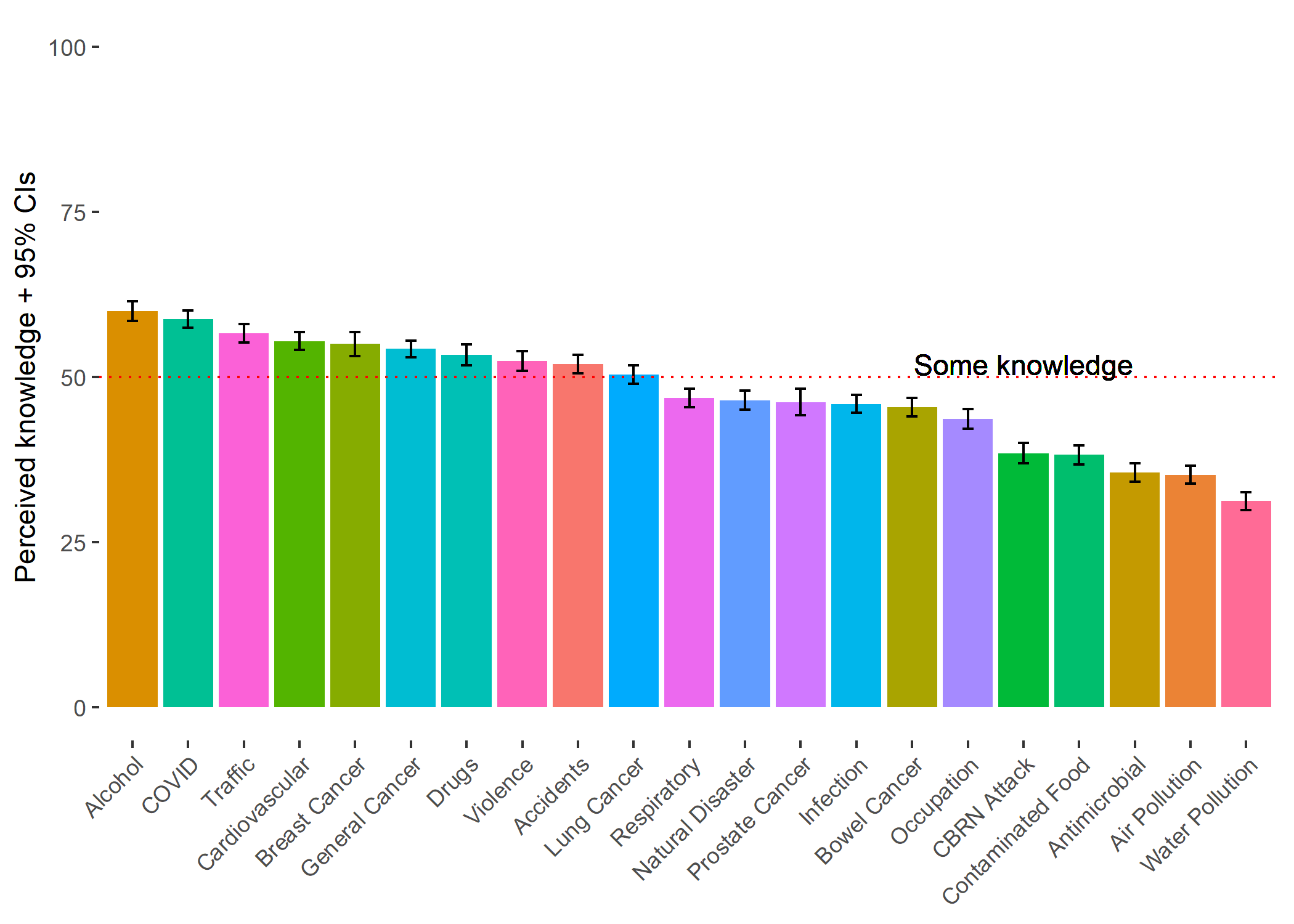


**Figure S2.** Perceived knowledge of risk for each cause of death.

*^Bars represent mean scores out of 100 for each cause of death, with 95% confidence intervals. N = 1,463 for all causes except breast cancer (n = 742 females) and prostate cancer (n = 714 males).^*


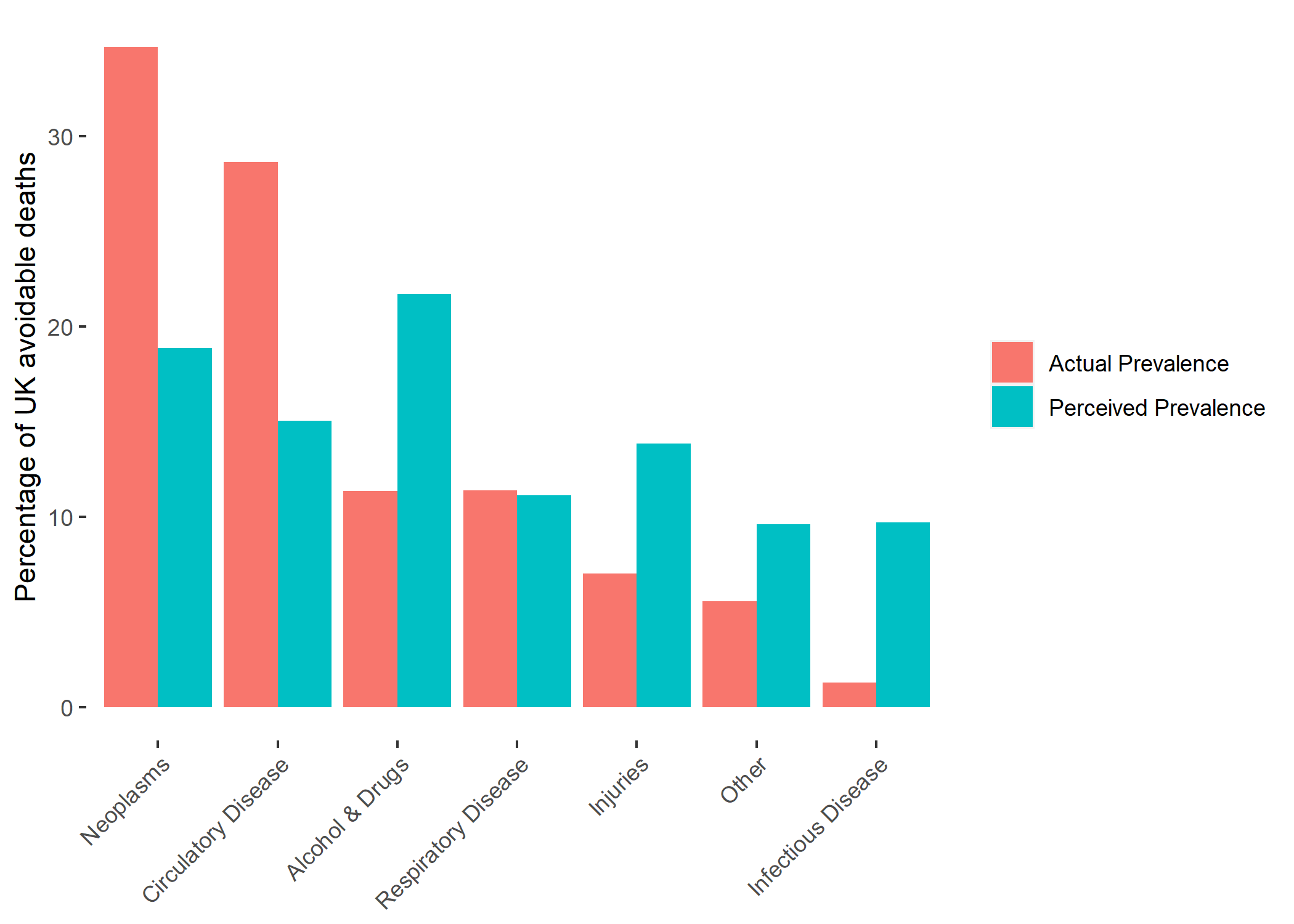


**Figure S3.** Comparison of perceived and actual prevalence of avoidable deaths in the UK.

**Table S1.** Regression analyses showing socio-demographic predictors of perceived uncontrollable mortality risk.

| Predictor | *b* | *b*  95% CI  [LL, UL] | *sr^2^* | *sr^2^*  95% CI  [LL, UL] | Fit |
| --- | --- | --- | --- | --- | --- |
| (Intercept) | 42.11** | [34.29, 49.93] |  |  |  |
| Age | 0.01 | [-0.06, 0.09] | .00 | [-.00, .00] |  |
| Gender(Woman) | -1.17 | [-3.20, 0.86] | .00 | [-.00, .01] |  |
| Education | 0.10 | [-0.20, 0.39] | .00 | [-.00, .00] |  |
| Subjective Discretionary Income | -0.73** | [-1.17, -0.29] | .01 | [-.00, .02] |  |
| Discretionary Income | -0.00** | [-0.01, -0.00] | .01 | [-.00, .02] |  |
| Perceived Neighbourhood Safety | -1.72** | [-2.66, -0.78] | .01 | [-.00, .03] |  |
| Self-reported Occupational Exposure | 0.08 | [-0.04, 0.19] | .00 | [-.00, .01] |  |
|  |  |  |  |  | *R^2^*  = .069** |
|  |  |  |  |  | 95% CI [.04,.09] |
|  |  |  |  |  |  |

*^Note. A significant b-weight indicates the semi-partial correlation is also significant. b represents unstandardized regression weights. sr2 represents the semi-partial correlation squared. LL and UL indicate the lower and upper limits of a confidence interval, respectively.
* indicates p < .05. ** indicates p < .01.^*

**Table S2.** Pearson correlations showing the relationships between perceived knowledge of leading causes of avoidable death in the UK and the accuracy of participant estimations of their prevalence.

| Cause of death | *r* | *p* |
| --- | --- | --- |
| Overall knowledge and accuracy | 0.05 | 0.05 |
| Infections | -0.08 | <0.01** |
| Neoplasms (cancers) | 0.06 | 0.01* |
| Cardiovascular disease | 0.11 | <0.01** |
| Respiratory disease | 0.02 | 0.39 |
| Accidents and Injuries | -0.02 | 0.51 |
| Drugs and Alcohol | -0.03 | 0.18 |

*^Note. N = 1,46. * indicates p < .05. ** indicates p < .01. A positive r value suggests that the more knowledge someone perceived they had about a specific cause of death, the more accurate their estimation of the proportion of avoidable deaths accounted for by this cause in the UK. This supplementary analysis was not included in our preregistration protocol but was suggested by an anonymous reviewer for the Journal of Public Health.^*
